# Intramuscular Myxoma: Results from the Largest European Single-Center Study—Clinical and Pathological Findings and Syndromal Associations

**DOI:** 10.3390/diagnostics16050684

**Published:** 2026-02-26

**Authors:** Katharina Trumm, Alonja Reiter, Tobias M. Ballhause, Karl-Heinz Frosch, Anna Duprée, Andreas M. Luebke, Matthias H. Priemel

**Affiliations:** 1 Department of Traumatology and Orthopedics, University Medical Center Hamburg-Eppendorf, 20251 Hamburg, Germany; 2 Department of Trauma Surgery, Orthopedics and Sports Traumatology, BG Hospital Hamburg, 21033 Hamburg, Germany; 3 Department of General, Visceral and Thoracic Surgery, University Medical Center Hamburg-Eppendorf, 20251 Hamburg, Germany; 4 Department of Pathology, University Medical Center Hamburg-Eppendorf, 20251 Hamburg, Germany

**Keywords:** intramuscular myxoma, soft tissue tumor, GNAS mutation, Mazabraud syndrome

## Abstract

**Objectives:** Intramuscular myxomas (IMMs) are rare benign soft tissue tumors arising within large skeletal muscles. Their etiology is incompletely understood, but they are frequently associated with mutations (e.g., GNAS) and may occur in syndromic conditions such as Mazabraud and McCune–Albright syndromes. This study retrospectively analyzed clinical, radiological, histopathological, and molecular features of IMMs, including syndromic associations. **Methods:** A retrospective analysis was performed on 41 patients diagnosed with IMM who underwent biopsy or surgical resection between September 2011 and September 2022. Clinical, imaging, histopathological, and molecular data were evaluated using descriptive statistics. **Results:** The cohort included 27 females and 14 males with a mean age of 52.8 years. The most common tumor location was the quadriceps femoris, followed by other thigh muscles. Most patients presented with mild symptoms due to slow tumor growth. MRI was performed in all but one case, with additional imaging in 12 patients. Radiological differential diagnoses commonly included soft tissue sarcoma and hematoma. Complete tumor resection was achieved in 90.2% of patients. Immunohistochemistry was performed in 78.0%, most frequently showing CD34 positivity. GNAS mutation analysis was conducted in 85.4% of cases and was positive in 57.1%. Complications occurred in 9.8%, and no recurrences were observed during follow-up. **Conclusion:** IMMs are rare benign tumors that can be reliably diagnosed using histology supported by immunohistochemistry and GNAS mutation analysis. Complete surgical resection provides excellent outcomes with a low risk of complications or recurrence. Mazabraud syndrome should be considered in patients with multiple or recurrent IMMs and GNAS mutations.

## 1. Introduction

Intramuscular myxomas (IMMs) are uncommon benign soft tissue tumors predominantly found within the large skeletal muscles. They typically manifest in adults between the ages of 40 and 60, with a higher incidence observed in women than men. There are approximately 0.11 cases per 100,000 individuals annually [1,2]. Due to their rarity, comprehensive studies investigating this condition are infrequent, with a majority of the recent literature consisting primarily of case reports [3,4,5,6,7,8,9,10,11,12,13,14,15,16,17,18,19,20,21,22,23,24,25,26,27,28,29,30,31,32,33,34,35,36]. The precise risk factors and underlying etiology of IMM remain incompletely understood. However, IMMs are frequently associated with a specific genetic mutation in the guanine nucleotide-binding protein alpha subunit (GNAS) gene. This mutation is associated with the development of Mazabraud syndrome, a rare condition characterized by the coexistence of IMMs and fibrous dysplasia, a benign bone disorder resulting in weakened and distorted bone structure [37,38,39,40]. Another rare genetic disorder, McCune–Albright syndrome, is also associated with GNAS mutations. This syndrome is characterized by the presence of fibrous dysplasia, café au-lait spots (pigmented skin patches), and precocious puberty [41]. Cases have been reported where IMMs occurred in the context of McCune–Albright syndrome, further highlighting the link between GNAS mutations and the development of IMMs [42].

The clinical presentation of IMM is marked by its considerable variability and is not dependent on genetic mutations. Numerous patients describe the existence of a gradual, painless, palpable mass, while in certain cases, the tumor is fortuitously discovered during imaging procedures conducted for unrelated reasons. Pain is uncommon and typically associated with larger tumor sizes [18,43,44,45,46,47]. However, differential diagnoses for IMMs encompass various benign tumors, non-neoplastic conditions, and malignant tumors, as they can display comparable clinical and imaging characteristics [35,48].

Surgical resection is the established treatment approach for IMMs, and marginal excision is advised after histopathological confirmation through biopsy [2,43,46,49,50,51]. However, managing IMMs in patients with Mazabraud syndrome, who often present with multiple tumors, is challenging due to a potential increased risk of multiple tumor formation compared to individuals without the syndrome [37,38,39,40].

The objective of this study is to conduct the largest European retrospective analysis of IMMs and describe them by means of descriptive statistics. Moreover, IMMs’ association with syndromes is investigated, examining the clinical and pathological findings and syndromal associations and conducting a thorough review of the literature on IMM.

## 2. Patients and Methods

The study design involved a retrospective review of medical charts, focusing on patients who had undergone a biopsy or surgical resection for IMM between September 2011 and September 2022. The data analyzed in this study were derived from electronic medical records of patients diagnosed with IMM who received treatment at a certified sarcoma center affiliated with a university hospital.

In the analysis of all cases, the tissue specimens were processed and examined at the Department of Pathology. This ensured standardized histopathological evaluation for accurate diagnosis and assessment of IMMs. To analyze the collected data, descriptive statistics were employed, with mean values, standard deviation (SD), *p*-values, and percentages calculated using Excel (Version 2019, Microsoft, Redmond, WA, USA).

In addition to the data analysis, a comprehensive literature review was conducted to gather relevant information on IMM. The narrative review encompassed all available literature on IMM since its initial description. Therefore, the PubMed database was used as the primary source for identifying and analyzing relevant studies. Additionally, related sources cited in the identified primary literature were included.

The study protocol was reviewed and approved for retrospective listing by the Regional Ethics Committee (WF-066/21). The need for individual patient consent was waived by the Ethics Committee in its decision.

## 3. Results

### 3.1. Demographic Data

**Figure 1:**
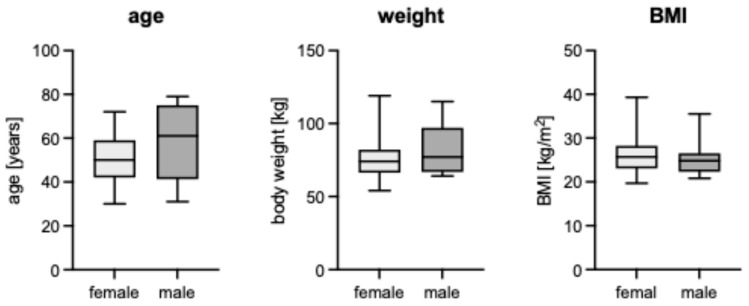
Comparison of age (years), weight (kg), and BMI (kg/m^2^) between female and male patients with intramuscular myxoma.

A total of 41 patients were included in the analysis, comprising 27 females and 14 males. The mean age at the time of diagnosis was 52.8 years, ranging from 30.0 to 79.0 years (SD: 13.4 years). The mean age of male patients was 57.6 years compared to 50.4 years in female patients, without a statistically significant difference (*p* = 0.16) (Figure 1).

The height of the patients ranged from 158.0 cm to 196.0 cm, with an average height of 173.4 cm (SD: 9.8 cm). Regarding weight, the patients had a weight range of 54.0 kg to 119.0 kg, with a mean weight of 78.4 kg (SD: 16.3 kg). The mean body mass index (BMI) was calculated as 26.0 kg/m^2^, with a range of 19.7 kg/m^2^ to 39.3 kg/m^2^ (SD: 4.5 kg/m^2^). There were no statistically significant differences between male and female patients regarding weight (*p* = 0.24) or BMI (*p* = 0.40). Furthermore, the cohort was predominantly Caucasian.

Among the patient population, 19.5% had a history of cancer, while 14.6% had a history of another benign tumor. The most common comorbidities observed were thyroid diseases in 29.3% of the patients, followed by varicosis in 22.0% and hypertension in 19.5% (Table 1).

### 3.2. Anatomical Distribution of IMMs

The majority (51.2%) of the analyzed IMMs were found in the proximal thigh, indicating a predilection for this region. Distal leg muscles, such as the M. tibialis anterior or the M. gastrocnemius, accounted for 12.2% of cases. Additionally, 12.2% of IMMs were located in the Mm. glutei, while 17.1% involved upper body muscles such as the M. deltoideus or the M. triceps brachii. Forearm involvement was observed in 2.4% of cases, and 4.9% occurred in the autochthonous back muscles.

### 3.3. Clinical Presentation, Diagnostic Challenges, and Imaging Modalities of IMMs

At the time of diagnosis, most patients presented with mild symptoms. Approximately 63.4% of cases exhibited an asymptomatic, painless, palpable mass or swelling, which commonly served as the initial clinical manifestation. However, in one-third of these cases, symptoms such as pain, local tenderness, or a tingling sensation developed later. In 26.8% of cases, patients reported pain during movement, walking, or pressure sensitivity. However, only 9.8% of patients were completely asymptomatic, reporting neither pain nor palpability. In these instances, the tumors were incidentally detected during MRI, CT, or phlebology examinations. At diagnosis, the mean diameter measured at the longest extent of IMM was 4.9 cm (SD: 2.1 cm) in diameter.

The suspected diagnoses for IMMs varied between radiological and clinical examinations. Common radiological diagnoses included soft tissue sarcoma and hematoma. Clinically, schwannoma and sarcoma were frequently considered in the differential diagnosis. Magnetic resonance imaging (MRI) played a crucial role in the evaluation of IMMs, with its utilization in 97.6% of cases (except for one case with contraindications). Complementary imaging techniques, including CT, PET-CT, ultrasonography, and/or radiography, were performed in 29.3% of cases.

**Figure 2:**
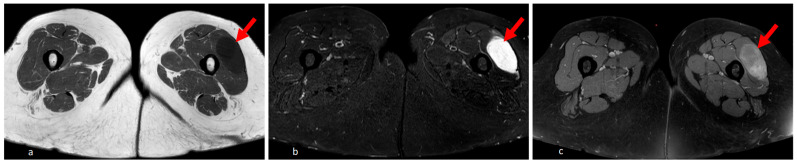
Axial MRI images of an intramuscular myxoma in the vastus lateralis muscle. The lesion appears well-defined and hypointense on the T1-weighted image (**a**), hyperintense on the T2-weighted sequence (**b**), and shows inhomogeneous enhancement after contrast administration (**c**). The arrow highlights the intramuscular myxoma.

On MRI, IMMs presented as smooth-edged intramuscular masses without infiltration of surrounding structures. They exhibit varying degrees of signal homogeneity, typically appearing hypointense on T1-weighted images and hyperintense on T2-weighted and fat-saturated sequences. After the administration of contrast medium, IMMs often displayed inhomogeneous enhancement (Figure 2).

### 3.4. Histopathology, Immunohistochemistry, and Molecular Pathology

The histopathological results were obtained from both biopsy and resection specimens. In the majority of cases (56.1%), an open biopsy was conducted prior to resection. The remaining cases (34.1%) underwent primary excision biopsy. Despite the recommended course of tumor resection following biopsy results, four patients opted not to proceed with surgical intervention. Overall, 90.2% of patients underwent tumor resection.

**Figure 3:**
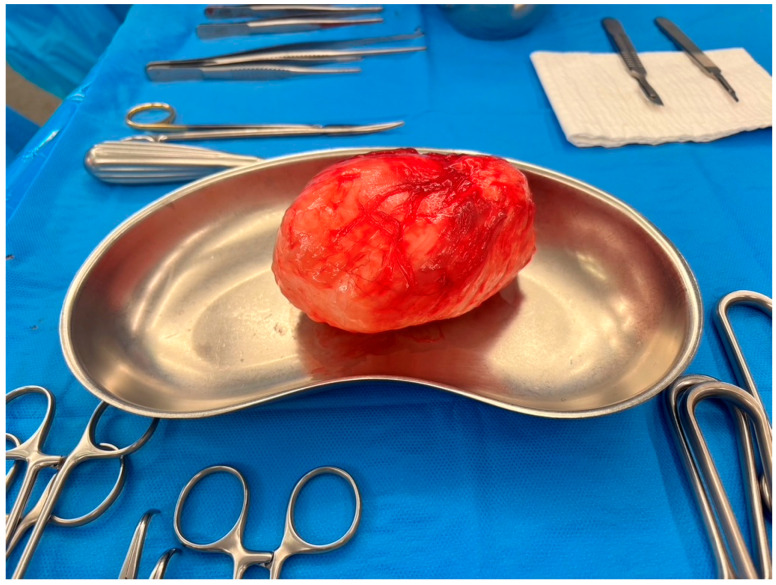
Macroscopic view of intramuscular myxoma: the classic intramuscular myxoma is typically well-defined and commonly encapsulated by a surrounding fibrous capsule. This image shows an intramuscular myxoma, which was resected from the m. vastus lateralis. The tumor measures 12.6 × 10.5 × 6 cm.

Macroscopically, the IMMs exhibited characteristics of a firm-elastic to gelatinous mass with myxoid or mucinous features (Figure 3). The tumors displayed varying shades of white, beige, or grey coloration, along with a white, beige, or grey and glassy cut surface. Cystic, slimy, or hemorrhaged areas were occasionally observed, and the tumors were pervaded by septa. In many cases, the IMMs were delicately membrane-enclosed and sharply demarcated.

**Figure 4:**
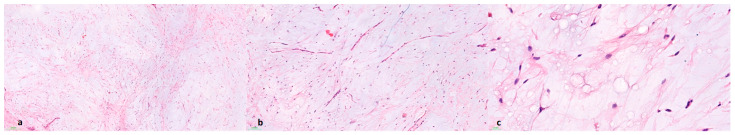
Microscopic view of an intramuscular myxoma: (**a**) the classic intramuscular myxoma is a hypocellular tumor composed of cytologically bland, uniform spindle cells embedded in a myxoid stroma (HE, 50×). (**b**) Delicate capillary-sized vessels are distributed throughout the tumor (HE, 100×). (**c**) At higher magnification, inconspicuous fibroblasts and myofibroblasts with uniform, oval-shaped nuclei become visible. Vacuoles can be seen within the myxoid stroma (HE, 400×).

Microscopically, the IMMs appeared as myxoid spindle-cell tumors without any cytomorphological atypia (Figure 4). The cell content ranged from scanty to relatively cell-rich, with cells exhibiting small, ovoid nuclei and partly eosinophilic cytoplasm. No evidence of malignancy was detected in any of the cases. Immunohistochemical examinations were conducted in 78.0% of the samples. The most frequently examined markers included AE1/AE3, CD34, Desmin, EMA, SMA, S100, and Mib-1/Ki-67. Other markers such as MUC4 and STAT6 were analyzed in only a few cases. CD34 was the most commonly positive marker, observed in 89.7% of the IMMs tested for this marker, followed by SMA (26.9%) and Desmin (21.4%). AE1/AE3 and S100 were negative in all analyzed samples. The proliferation marker Ki-67 marked less than 1.0 to 5.0% of the tumor cells. In terms of molecular pathological examination, 85.4% of the IMMs were tested for GNAS mutations. Among these, 57.1% showed a positive GNAS mutation and 42.9% were negative. The most prevalent mutation type was c.602G > A; p.R201H, accounting for 70.0% of all GNAS mutations. Other mutation types included c.601C > T; p.R201C, c.601C > A; p.R201S, and c.602G > T; p.R201L. Molecular examinations for sarcoma markers were performed in 29.3% of cases, including DDIT3 (CHOP) translocation, FUS translocation, and MDM2 gene amplification, all of which yielded negative results in IMMs (Supplementary Table S1).

### 3.5. Treatment and Complications

The primary therapeutic approach for IMMs in our study involved achieving complete resection. In all cases, resection was performed in total with no tumor margins left in situ. None of the patients required additional therapies, such as radiation or systemic treatment. Thus, total tumor resection is an appropriate and stand-alone treatment option for IMMs.

In terms of complications, they were observed in a relatively low proportion of cases, specifically 9.8%. These complications encompassed a hematoma after open biopsy, a wound seroma, a hematoseroma, and a wound infection. In half of the cases, surgical intervention was required to address the respective complications. It is worth noting that although these complications occurred in a small subset of patients, they were managed appropriately and did not lead to any adverse outcomes or affect the long-term prognosis of the individuals.

## 4. Discussion

### 4.1. Epidemiology and Etiology

IMMs commonly affect middle-aged adults and exhibit a higher prevalence in women compared to men [44]. In line with previous studies, our research confirms this gender disparity. Among the reported tumor locations, the muscles of the thigh were most frequently affected, followed by the gluteal muscles and muscles of the shoulder, upper arm, and lower leg [46,47,49,50,52]. These findings align with the prevailing literature, emphasizing the typical anatomical sites associated with this tumor. Our study differs from Enzinger’s previous findings, which reported no age difference between men and women [43]. In contrast, our analysis showed a tendency for male patients to be older than female patients, although this difference was not statistically significant.

The etiology of IMMs remains unclear, and although various causes have been discussed, such as previous trauma or hypothyroidism, no specific risk factors have been identified [43,44,46]. In our patient cohort, a history of trauma in close proximity to the tumor was reported in only a small percentage of cases. Hypothyroidism or a history of thyroidectomy was reported in 29.3% of cases, which is substantially higher than the prevalence reported in international cohort studies [53,54]. However, it is important to note that the observed higher prevalence does not establish a clear correlation between IMM and thyroid dysfunction or thyroidectomy. The relationship between these factors requires further research and investigation to determine if a true association exists. An additional risk factor for IMMs that has been discussed in the literature is a history of radiation exposure [55]. However, in our patient collective, none of the cases reported prior radiation exposure near the IMM.

In one case, Mazabraud syndrome was diagnosed, and in one other case, there was a suspicion of Mazabraud syndrome because multiple soft tissue tumors in the M. vastus medialis were detected in MRI, which is a rare occurrence. However, two of the three masses could not be detected intraoperatively, so cystic lesions were assumed. In addition, there was no evidence of fibrous dysplasia. Mazabraud syndrome is characterized by the combination of IMMs and fibrous dysplasia, and long-term follow-up should be performed in such cases to monitor for potential malignancy [37,38,39,40]. In the largest study on Mazabraud syndrome, a long-term follow-up was performed in 93.75% of cases [37]. In most cases, the diagnosis of Mazabraud syndrome is made very late, on average about 10 years after the diagnosis of fibrous dysplasia. Fibrous dysplasia is typically detected on radiography, while IMMs are best visualized on MRI. In almost all cases, a GNAS mutation occurs in fibrous dysplasia as well as in IMMs [37,38].

### 4.2. Diagnostic Challenges

In the majority of cases, patients presented with mild symptoms. A common complaint among many patients was the presence of a painless palpable mass, which is also documented as the most frequently reported symptom in the existing literature [43,44,47,50]. These mild symptoms can often be attributed to the typically slow growth pattern of IMMs [46]. However, in isolated cases, rapid initial growth [43] or a change in size may occur after several years of stability [44].

Overall, magnetic resonance imaging (MRI) appears to be the preferred diagnostic modality for evaluating soft tissue tumors, including IMMs. In our series, MRI was performed in nearly all patients before undergoing biopsy or resection. It is noteworthy that our patients presented with diverse preoperative diagnoses, and there was often a lack of consensus between radiologists and surgeons regarding a definitive diagnosis. Accurately diagnosing IMMs is challenging and frequently necessitates histopathological examination of tissue, as previous studies have indicated [43,44,47,50,56].

### 4.3. Histopathological Features and Molecular Markers

Following clinical examination and imaging, all of our patients underwent histopathological confirmation of the diagnosis, either through biopsy or primary resection, in accordance with the national guideline [51]. However, where possible, it is recommended to perform an open biopsy before resection. This approach can confirm the preoperative diagnosis of a malignant tumor, aid in surgical planning, and prevent inadequate marginal resection. In cases of benign tumor entities, it can also prevent unnecessarily wide resections that may lead to significant loss of function. Enzinger reported a case where a leg amputation was performed due to a misinterpretation of the tumor as myxoid liposarcoma, highlighting the importance of accurate diagnosis [43]. In cases with a high perioperative risk of complications or when the tumor is difficult to access for biopsies due to anatomical conditions, direct resection may be necessary.

The macroscopic and microscopic findings of IMMs in this study agree with the results in the literature. There, the tumor is also described as a white or gray, myxoid intramuscular mass with a gelatinous and glassy cut surface and some cystic or slimy spaces. The cells are spindle or stellate shaped in a myxoid matrix and have no cytomorphological atypia [44,46,49,50,57,58,59].

Molecular and immunohistochemical markers play a central role in confirming the diagnosis of IMM. These examinations contribute to the differentiation of IMMs from other potentially malignant tumors, such as low-grade myxofibrosarcoma or low-grade fibromyxoid sarcoma, which are histologically similar [57,58,59,60]. In this study, CD34 was the most frequently positive marker. CD34 is a sensitive, although not specific, immunohistochemical marker for IMMs and is positive in the majority of cases [57,59]. Other immunohistochemical markers were rarely or not expressed, consistent with previous studies [49,59]. GNAS mutation analysis revealed mutations in 57.1% of the analyzed IMMs, with c.602G > A; p.R201H being the most common variant. In contrast to many other tumors, GNAS mutations are frequently detected in IMMs, most commonly involving the c.602G > A; p.R201H mutation. While the reported sensitivity of GNAS mutations varies and is approximately 68.0 to 92.3%, their specificity for IMM is considered high [57,58,59,60]. Therefore, the combined evaluation of GNAS mutation status, especially the detection of p.R201H, and CD34 expression constitutes a valuable diagnostic approach and enhances the differentiation of IMMs from other tumor entities.

### 4.4. GNAS Mutation and Mazabraud Syndrome

In Mazabraud syndrome, IMMs and fibrous dysplasia are associated with mutations in the GNAS gene [37]. In our study, a GNAS mutation was identified in 57.1% of the IMMs analyzed. However, as GNAS mutations frequently occur in sporadic IMMs, their presence alone does not suggest Mazabraud syndrome. Both IMMs and fibrous dysplasia often present asymptomatically [38,39]. Typically, fibrous dysplasia is diagnosed long before the detection of IMMs, but the rare Mazabraud syndrome is often diagnosed after the identification of an IMM [37,39,40]. IMMs in these contexts are usually multiple or recurrent [38,39,40]. Cases of recurrent IMM have highlighted delays in the diagnosis of fibrous dysplasia and Mazabraud syndrome, potentially leading to deformities, fractures, or pain. Fibrous dysplasia also carries a low risk of malignancy [37,40,61].

Given the frequent anatomical co-localisation of IMMs and fibrous dysplasia, it may be reasonable for patients with multiple or recurrent IMMs to undergo radiographic evaluation of the affected region to rule out fibrous dysplasia and Mazabraud syndrome [37,38]. In contrast, additional radiographic screening may not be necessary in solitary IMMs, even in the presence of a GNAS mutation [37,38,39,40].

### 4.5. Treatment Options for IMMs and Therapeutic Concepts in Mazabraud Syndrome

Although wait-and-watch could be a potential option for asymptomatic cases, the majority of IMMs tend to grow over time and can lead to pain and movement restrictions. In some cases, the tumor may even cause impairment of adjacent structures, such as nerves or blood vessels. Therefore, a more aggressive surgical intervention may be required if the tumor becomes symptomatic or continues to grow [2,43,44,46].

Non-surgical therapies such as radiotherapy or systemic therapy are not effective in treating benign tumors as IMMs. Therefore, the primary treatment option remains surgical resection, which is usually associated with a low risk of complications. Achieving complete tumor resection is crucial in preventing recurrences and is considered the most significant prognostic factor for IMMs [1,2,43,44,45,46,49,50]. In our study, we successfully achieved complete tumor resection in all patients, and no further therapy was required. However, there is limited literature specifically addressing the optimal treatment approach for multiple IMMs in Mazabraud syndrome; some insights can be obtained from the available studies. Multiple case reports and case series have described surgical resection as the primary treatment modality for symptomatic IMMs associated with Mazabraud syndrome. Complete resection aims to remove all visible tumors while preserving the function of affected muscles. It is important to note that the decision for surgical intervention should be individualized based on factors such as the size, location, symptoms, and potential impact on adjacent structures.

Furthermore, no local recurrences were observed in our cohort following complete surgical resection. Given the absence of cellular atypia or other malignant features in our series, true invasive behavior appears unlikely. By definition, IMM is a benign and non-infiltrative tumor. Unlike IMM, malignant myxoid tumors such as myxoid fibrosarcoma or myxoid liposarcoma represent important differential diagnoses and are associated with a substantial risk of local recurrence and metastasis due to their infiltrative growth patterns [62,63]. In contrast, syndromic associations, particularly Mazabraud syndrome, have been associated with higher reported recurrence rates. In this context, recurrence is more likely related to multifocal tumor development and the underlying GNAS-driven pathogenesis accompanying fibrous dysplasia rather than true invasive growth [37]. Therefore, long-term follow-up, especially in syndromic cases, is advisable to detect potential recurrences. However, due to the retrospective study design, recurrence data were obtained passively and depended on patients reporting to our institution. Therefore, asymptomatic recurrences or cases managed elsewhere cannot be entirely excluded.

### 4.6. Limitations

When interpreting the results of this study, it is important to consider several limitations. Firstly, the rarity of IMMs restricts the availability of literature, resulting in limited data comprising small case series and individual case reports. This scarcity hampers comprehensive analysis and generalizability of findings. Secondly, treatment decisions were tailored on a case-by-case basis, lacking a standardized treatment pathway. This individualized approach introduces inherent variability in treatment outcomes, making it challenging to establish definitive guidelines. Although this is one of the largest studies on IMMs, due to the rarity of the tumors, the sample size is restricted.

## 5. Conclusions

IMMs are rare soft tissue tumors and an important differential diagnosis to sarcomas. The etiology and pathogenesis of IMMs are still incompletely understood. Although certain risk factors have been identified, including gender, chronic diseases, and the presence of Mazabraud syndrome, the precise mechanisms underlying their development remain unclear. Pathological examination, including immunohistochemical analysis for CD34 and GNAS mutation testing, plays a crucial role in confirming the diagnosis and distinguishing IMMs from other neoplastic entities. In patients with multiple or recurrent IMMs in combination with a GNAS mutation, Mazabraud syndrome should also be considered. In cases of Mazabraud syndrome, where multiple tumors may occur, the indication for resection should be carefully considered. Complete marginal resection of IMMs remains the preferred treatment approach, with a favourable prognosis and low risk of complications or recurrence. Further research is needed to elucidate the molecular mechanisms and to better understand the syndromic associations of IMMs in order to improve diagnostic and therapeutic strategies.

## Figures and Tables

**Table 1:** Demographic Characteristics of Patients with Intramuscular Myxoma. This table summarizes the baseline demographics. Variables include age, sex, height, weight, and body mass index (BMI) at the time of diagnosis, as well as relevant comorbidities and potential risk factors.

Case	Age	Sex	Height (cm)	Weight (kg)	BMI (kg/m^2^)	Comorbidities	Profession	Anatomic Location
1	33	F	178	82.0	25.9	uterine myoma, hypothyroidism, re-entry tachycardia	scientist	*M. vastus lateralis*
2	40	F	174	119.0	39.3	obesity, hypothyroidism, hypertension, arrhythmia, bronchial asthma, lipedema, hepatic steatosis, craniomandibular dysfunction	online marketing manager	*M. vastus lateralis*
3	63	F	165	94.0	34.5	obesity, hypertension, varicosis, bronchial asthma, chronic bronchitis, gallstones, kidney stones, cataract, arthrosis, disc prolapse	seller	*M. vastus lateralis*
4	79	M	173	73.0	24.4	gout, cataract	pensioner	*M. vastus lateralis*
5	33	M	169	64.0	22.4	-	goldsmith	*M. vastus lateralis*
6	53	F	176	61.0	19.7	breast cancer, varicosis	office clerk	*M. vastus medialis*
7	68	F	182	75.0	22.6	breast cancer, melanoma in situ, mycosis fungoides, Bowen’s disease, actinic keratosis, basalioma, thyroidectomy, cataract	unknown	*M. vastus medialis*
8	62	M	196	95.0	24.7	hypertension, acute coronary syndrome, atherosclerosis, type 2 diabetes, gallstones, urinary tract dysfunction	service technician	*M. vastus medialis*
9	67	F	172	63.0	21.3	hypertension	housewife	*M. vastus medialis*
10	65	M	167	70.0	25.1	-	unknown	*M. vastus medialis*
11	56	M	178	66.0	20.8	hypertension, kidney stones, history of tuberculosis	self-employed	*M. vastus medialis/intermedius*
12	37	F	190	92	25.5	breast fibroadenoma, hypothyroidism, alpha-1 antitrypsin deficiency, varicosis, urticaria	housewife	*M. vastus medialis/intermedius*
13	38	F	165	73.0	26.8	hypothyroidism, gallstones	doctor	*M. vastus intermedius*
14 *	75	M	172	67.0	22.6	prostate cancer, colon polyps, hypertension, coxarthrosis, spondylolisthesis	pensioner	*M. vastus intermedius*
15	55	F	172	80.0	27.0	uterine myoma	educator	*M. pectineus*
16	75	M	190	110.0	30.5	obesity, glaucoma	pensioner	*M. adductor magnus*
17	31	M	188	93.0	26.3	-	footballer	*M. semitendinosus*
18	50	F	167	68.0	24.4	-	commercial clerk	*M. gluteus maximus/M. semitendinosus*
19	55	F	162	54.0	20.6	hypothyroidism, cataract	unknown	*M. gluteus maximus*
20	57	F	178	115.0	36.3	obesity	employee	*M. gluteus maximus/medius*
21	30	F	160	56.0	21.9	bronchial asthma, tinnitus, migraine	bank clerk	*M. gluteus medius*
22	62	F	163	75.0	28.2	lipoma, bronchoadenoma, bronchial asthma	professor	*M. tibialis anterior*
23	45	F	170	63.0	21.8	-	assistant	*M. tibialis anterior*
24	75	M	180	81.0	25.0	hypertension, varicosis, macular degeneration	retired lecturer	*M. gastrocnemius*
25	49	F	163	70.0	26.3	-	saleswoman	*M. gastrocnemius medialis*
26	59	F	158	70.0	28.0	breast cancer, uterine myoma, ovarian cysts, hypothyroidism, multiple sclerosis, depressions	housewife	*M. soleus*
27	47	F	170	68.0	23.5	breast cancer, Graves’ disease (subtotal thyroidectomy), varicosis	seller	*M. deltoideus*
28	49	F	170	67.0	23.2	hypothyroidism, stroke, pulmonary artery embolism, depressions	hotel manager	*M. deltoideus*
29	53	M	184	73.0	21.6	arrhythmia	editor	*M. deltoideus*
30	52	F	165	77.0	28.3	rectal cancer, uterine myoma, ovarian cysts, endometriosis, hepatopathy, therapy-indicated polyneuropathy	pharmaceutical consultant	*M.trapezius/M. suprascapularis*
31	43	F	161	64.0	24.7	uterine myoma, varicosis, bronchial asthma	commercial clerk	*M. triceps brachii*
32	60	M	195	103.0	27.1	factor V-Leiden mutation, deep vein thrombosis, arrhythmia, varicosis, cataract, ablatio retinae, coloboma left eye, disc prolapse, depressions	scientist	*M. triceps brachii*
33	44	F	162	82.0	31.2	lipoma, obesity, varicosis, gallstones	housewife	*M. triceps brachii*
34	72	F	165	67.0	24.6	breast cancer, hemorrhagic diathesis, varicosis, chronic hepatitis C, cataract, macular degeneration, obstructive sleep apnea syndrome, chronic sinusitis, osteoporosis, rhizarthrosis	pensioner	*forearm*
35	33	M	170	64.0	22.1	-	teacher	*autochthonous back muscles*
36	34	F	168	90	31.9	hypothyroidism, glaucoma, multiple eosinophilic granulomata, bronchial asthma	n.r.	*M. vastus lateralis*
37	55	F	n.r.	n.r.	n.r.	hypothyroidism, hypertension	n.r.	*M. adductor magnus*
38	65	M	186	86	24.9	-	n.r.	*Mm. glutei*
39	61	F	180	76.0	23.5	amelanotic melanoma, arthrosis	librarian	*M. vastus lateralis*
40	44	M	180	115.0	35.5	obesity, hypothyroidism	n.r.	*dorsal thigh*
41	42	F	170	75.0	26.0	-	doctor	*M. deltoideus*

M = male; F = female; BMI = body mass index; * Mazabraud syndrome.

## Data Availability

The datasets used during the current study are available from the corresponding author upon reasonable request.

## Supplementary Materials

The following supporting information can be downloaded at: https://www.mdpi.com/article/10.3390/diagnostics16050684/s1, Supplementary Table S1: Macroscopical, microscopical, immunohistochemical and molecular pathological findings.
