# Supplementary material for: Intramuscular Myxoma: Results from the Largest European Single-Center Study—Clinical and Pathological Findings and Syndromal Associations
Source: Diagnostics (Basel). 2026 Feb 26;16(5):684. doi: 10.3390/diagnostics16050684 (PMC12984141; doi:10.3390/diagnostics16050684)
Supplement: Supplementary file 1 [file diagnostics-16-00684-s001.zip › Supplementary Table.pdf]

Supplementary Table S1: Macroscopical, microscopical, immunohistochemical and molecular pathological findings

Case no.		Macroscopic findings	Microscopic findings	Immunohistochemistry								Molecular pathology			
				AE1 / AE3	CD34	Desmin	EMA	SMA	S100	Mib-1 / Ki-67	Other	GNAS1 mutation	DDIT3 (CHOP) translocation	FUS translocation	MDM2 gene amplification
**1**	open biopsy	whitish, firm-elastic to gelatinous	myxoid, spindle-cell tumor	negative	positive *	n.r.	negative	positive *	negative	< 1%	MUC4: negative	yes [2]	-	-	-
	resection	beige-white, firm-elastic, delicately membranous enclosed<br>cut surface: white, glassy, mucinous	n.r.	-	-	-	-	-	-	-	-	-	-	-	-
**2**	open biopsy	beige, glassy-gelatinous, fragmented	myxoid, spindle-cell tumor	negative	positive *	negative	negative	negative	negative	< 1%	-	yes [2]	-	-	-
**3**	open biopsy	gray, myxoid, irregularly edged	relatively cell-rich, myxoid, spindle-cell tumor	negative	positive	negative	positive *	negative	negative	1 %	MUC4: negative	yes [2]	no	no	-
	resection	beige-gray, membrane-enclosed<br>cut surface: beige-gray, gelatinous, partly solid, partly cystic	n.r.	-	-	-	-	-	-	-	-	-	-	-	-
**4**	open biopsy	gray, glassy, partly gelatinous	mesenchymal, myxoid, spindle-cell tumor	negative	positive *	negative	negative	negative	negative	< 5%	MUC4: negative	no	no	no	-
	resection	brown-gray, firm-elastic<br>cut surface: multicystic, slimy	myxoid, spindle-cell tumor	-	-	-	-	-	-	-	-	-	-	-	-
**5**	open biopsy	white, gelatinous	myxoid, spindle-cell tumor	negative	positive	positive *	negative	positive *	negative	< 5%	-	yes [2]	-	no	-
	resection	gelatinous, sharply demarcated	n.r.	-	-	-	-	-	-	-	-	-	-	-	-
**6**	open biopsy	myxoid	mesenchymal, myxoid, spindle-cell tumor with small, ovoid cell nuclei and partly eosinophilic cytoplasm	-	negative	positive *	-	-	negative	4 %	-	yes	-	-	-
	resection	gray-white, mostly slimy, sharply demarcated,<br>partly slightly hemorrhaged	focal cell-rich tumor	-	-	-	-	-	-	-	-	-	-	-	-
**7**	excision biopsy	almost homogeneous gray-glassy, firm-elastic, sharply demarcated, cystic area in the edge area	myxoid tumor	-	-	-	-	-	n.r.	n.r.	MUC4: n.r.	no	no	no	-
**8**	open biopsy	gelatinous	mesenchymal, myxoid, spindle-cell tumor	negative	positive *	positive *	negative	positive *	negative	1 %	-	yes [2]	-	-	-

	resection	gray-white, gelatinous, sharply demarcated, delicately encapsulated	n.r.	-	-	-	-	-	-	-	-	-	-	-	-
**9**	open biopsy	white, myxoid, elastic	myxoid, spindle-cell tumor	negative	positive *	negative	negative	negative	negative	1 %	MUC4: n.r.<br>STAT6: n.r.	no	no	no	no
	resection	whitish, gelatinous, sharply demarcated	myxoid, spindle-cell tumor	negative	positive	negative	negative	positive *	negative	< 5%	-	-	-	-	-
**10**	open biopsy	n.r.	n.r.	n.r.											
	resection	n.r.	n.r.	n.r.											
**11**	open biopsy	gray-white, gelatinous, elastic	myxoid, spindle-cell tumor	negative	positive	negative	negative	negative	negative	< 1%	-	yes [2]	-	-	-
	resection	delicately encapsulated<br>cut surface: light beige, glassy, finely lobulated, yellowish-glassy parts and focal hemorrhages on the edge	n.r.	-	-	-	-	-	-	-	-	-	-	-	-
**12**	open biopsy	gray-beige, glassy, rugged	myxoid, spindle-cell tumor	negative	positive *	negative	negative	negative	negative	< 1%	MUC4: negative	no	-	-	-
	resection	gray-beige, firm-elastic<br>cut surface: gray-beige, myxoid, slimy	n.r.	-	-	-	-	-	-	-	-	-	-	-	-
**13**	excision biopsy	largely homogenous light-beige, myxoid, indicated swirling, partly loosened, sharply demarcated, delicately encapsulated	myxoid, spindle-cell tumor	negative	positive *	negative	negative	positive *	negative	1 %	-	no	-	-	-
**14**	open biopsy	white, gelatinous	cell-poor, myxoid, spindle-cell tumor, surrounded by a thin pseudocapsule	-	-	-	-	-	-	-	-	yes [2]	-	-	-
**15**	core-needle biopsy	whitish, frayed	n.r.	-	-	-	-	-	-	-	-	-	-	-	-
	open biopsy	yellowish-reddish, glassy with a pseudocystic, gelatinous aspect	cell-poor, lobulated, myxoid tumor	-	-	-	-	-	-	-	-	yes [4]	-	-	-
	resection	myxoid, slightly encapsulated	myxoid, spindle-cell tumor	negative	positive	negative	-	-	negative	< 1%	-	no	-	-	-
**16**	excision biopsy	white-gray, mucinous	partially cell-rich tumor	-	-	-	-	-	-	-	-	-	-	-	-
**17**	open biopsy	gray-white, glassy	cell-poor, myxoid, spindle-cell tumor	n.r.	positive	negative	negative	positive *	negative	n.r.	-	no	-	no	-

	resection	whitish-shiny, firm-elastic cut surface: homogenous light yellow-whitish, with a reddish hemorrhage	myxoid, monomorphic, spindle-cell tumor	negative	positive	positive *	negative	negative	negative	1-2 %	MUC4: negative STAT6: positive *	-	-	-	-
**18**	excision biopsy	knotty, firm-elastic cut surface: cystic with clear, viscous secretion	myxoid, spindle-cell tumor	negative	positive *	negative	negative	negative	negative	< 1%	-	no	-	-	-
**19**	excision biopsy	intact encapsulated cut surface: homogenous white, myxoid, focal cystic, partly slimy	myxoid, spindle-cell tumor	-	positive	positive *	-	positive *	negative	-	-	yes [2]	-	-	-
**20**	open biopsy	white, firm-elastic, partly slimy	myxoid, spindle-cell tumor	negative	negative	positive *	negative	positive *	negative	< 1%	-	no	no	no	no
	resection	gray-beige, partly reddish, partly lipomatous cut surface: whitish, firm-elastic, partly cystic	myxoid, spindle-cell tumor	-	-	-	-	-	-	-	-	-	-	-	-
**21**	open biopsy	glassy, myxoid	cell-poor, myxoid tumor with a few, small, fibroblast-like cells	-	-	-	-	-	-	-	-	-	-	-	-
	resection	myxoid, lobed, intact encapsulated cut surface: discreetly compressed white-yellowish towards the end, glassy, finely septate	n.r.	-	-	-	-	-	-	-	-	no	-	-	-
**22**	open biopsy	white-gray, firm-elastic, partly slimy	myxoid, spindle-cell tumor	negative	positive	negative	negative	negative	negative	< 1%	-	no	-	-	no
**23**	excision biopsy	gray-red-beige, predominantly myxoid and soft cut surface: gray-white, myxoid, partly cystically changed	myxoid, mesenchymal tumor	-	positive	negative	-	-	negative	< 1%	-	yes [2]	-	-	no
**24**	excision biopsy	beige-white, partly glassy, slimy	n.r.	-	-	-	-	-	-	-	-	-	-	-	-
**25**	excision biopsy	whitish, sharply demarcated cut surface: myxoid	cell-poor, myxoid, spindle-cell tumor	negative	negative	positive *	negative	negative	negative	< 1%	-	yes [2]	-	-	-
**26**	open biopsy	n.r.	cell-poor, myxoid, high vascular tumor with some enlarged, hyperchromatic cell nuclei	-	-	-	-	-	-	-	-	-	-	-	-
	resection	beige, glassy, soft	myxoid, spindle-cell tumor	negative	positive	negative	negative	negative	negative	< 1%	-	yes [1]	-	-	-
**27**	open biopsy	gray-white, gelatinous	myxoid tumor	-	-	-	-	-	-	< 0.1 %	MUC4: negative	yes [1]	-	-	-

	resection	gelatinous, multi-chambered, fuzzy-edged, pervaded by whitish septa	n.r.	-	-	-	-	-	-	-	-	-	-	-	-
**28**	open biopsy	reddish-white, soft	cell-poor, mesenchymal tumor	-	-	-	-	-	n.r.	n.r.	-	no	-	-	-
	resection	brownish-beige, firm-elastic, partly frayed, partly sinewy<br>cut surface: partly glassy, partly cystic	myxoid, spindle-cell tumor	negative	positive *	negative	negative	positive *	negative	5 %	STAT6: negative	-	no	-	-
**29**	open biopsy	gray-beige, soft, partly slimy	cell-poor, myxoid, spindle-cell tumor	negative	positive	negative	negative	negative	negative	1 %	-	no	-	-	-
**30**	excision biopsy	homogeneously whitish, gelatinous, solid, sharply demarcated and slightly encapsulated	myxoid tumor	-	-	-	-	-	-	-	-	no	-	-	-
**31**	open biopsy	white, partially transparent, slimy thread-pulling	myxoid, spindle-cell tumor	n.r.	n.r.	n.r.	n.r.	n.r.	n.r.	n.r.	MUC4: n.r.	yes [1]	-	no	-
	resection	firm-elastic, well-edged<br>cut surface: gray, glassy, septate	myxoid, spindle-cell tumor	-	-	-	-	-	-	-	-	-	-	-	-
**32**	open biopsy	gray-brown-beige, partly red, slimy	myxoid, spindle-cell tumor	negative	positive	negative	negative	negative	negative	< 1%	-	yes [2]	-	-	-
	resection	beige-gray, slightly encapsulated<br>cut surface: homogeneous gray-white, partly translucent	n.r.	-	-	-	-	-	-	-	-	-	-	-	-
**33**	open biopsy	white, glassy, slimy	myxoid, spindle-cell tumor	negative	positive	negative	negative	negative	negative	< 1%	-	yes [2]	-	-	-
	resection	gray, elastic<br>cut surface: gray, partly transparent, soft, myxoid aspects	n.r.	-	-	-	-	-	-	-	-	-	-	-	-
**34**	excision biopsy	beige-white, firm<br>cut surface: white, swirling	partly myxoid, partly spindle-cell tumor	negative	positive	negative	negative	negative	negative	< 1%	-	no	-	-	-
**35**	open biopsy	yellowish-whitish, partly gelatinous	partly myxoid, spindle-cell tumor	negative	positive	negative	negative	negative	negative	< 1%	MUC4: negative	yes [3]	-	-	no
	resection	nodular, partly cystic<br>cut surface: yellow, glassy, slimy	n.r.	-	-	-	-	-	-	-	-	-	-	-	-
**36**	excision biopsy	n.r.	n.r.	n.r.											
**37**	open biopsy	n.r.	n.r.	n.r.											

	resection	n.r.	n.r.	n.r.												
**38**	excision biopsy	n.r.	n.r.	n.r.												
**39**	open biopsy	white, glassy, partly slimy	myxoid, spindle-cell tumor	negative	positive	negative	negative	negative	negative	1 %	-	yes ²	-	-	-	
	resection	cut surface: myxoid, slimy	n.r.	-	-	-	-	-	-	-	-	-	-	-	-	
**40**	excision biopsy	smooth-shiny, slightly lipomatous<br>cut surface: white, myxoid, shiny	myxoid, spindle-cell tumor	negative	positive	negative	negative	negative	negative	1 %	-	no	-	-	-	
**41**	excision biopsy	gray-brown<br>cut surface: myxoid, slimy	myxoid, spindle-cell tumor	negative	positive	negative	negative	negative	negative	2 %	-	yes ²	-	-	-	

*** partially / in a few cells ¹ mutation c.601C>T; p.R201C ² mutation c.602G>A; p.R201H ³ mutation c.601C>A; p.R201S ⁴ mutation c.602G>T; p.R201L n.r. = not reported - = not performed**
